# Nausea control in mild head trauma patients: Effectiveness of metoclopramide and ondansetron in the emergency department in a double-blind study

**DOI:** 10.22088/cjim.13.4.699

**Published:** 2022

**Authors:** Hamideh Feiz Disfani, Mostafa Kamandi, Seyyedeh Bahareh Hoseini, Narges Shirazi, Maryam Panahi

**Affiliations:** 1 Department of Emergency Medicine, Faculty of Medicine, Mashhad University of Medical Sciences, Mashhad, Iran; 2 Department of Hematology Oncology, Faculty of Medicine, Mashhad University of Medical Sciences, Mashhad, Iran

**Keywords:** Mild head trauma, Nausea, Vomiting, Ondansetron, Metoclopramide

## Abstract

**Background:**

Mild head trauma often causes several complications and disabilities including nausea and vomiting in hospitalized people. The aim of the present study was to compare the effectiveness of metoclopramide and ondansetron, and compare it with placebo to control nausea in the patients with mild head trauma admitted to the emergency department.

**Methods:**

This is a randomized double-blind placebo-controlled clinical trial conducted on the patients with mild head trauma and normal brain CT scans who were admitted to the emergency department within 24 hours after the injury. The subjects were randomly divided into three groups of ondansetron (n= 41), metoclopramide (n= 44), and placebo (n= 39), and the severity of nausea and vomiting was assessed using the visual analogue scale (VAS).

**Results:**

A total of 124 patients with mild head trauma were included in the study. The assessment of the VAS scores during the study showed that over time, the patients in all three groups had reduced nausea (p<0.01). On the other hand, the percentage change of the VAS score indicated that metoclopramide and ondansetron had the greatest changes (46.97% and 66.90%, respectively) within 15 and 30 minutes after the injection, respectively.

**Conclusion:**

The results of the present study showed that ondansetron and metoclopramide had similar effects on nausea in the patients with mild head trauma. However, metoclopramide was most effective in 15 minutes and ondansetron in 30 minutes after the injection.

Nausea and vomiting are among the most common complaints of the patients admitted to the emergency department (1), affected by the factors such as sex (female), pregnancy, menstrual cycle, previous history of nausea and vomiting, smoking, duration of anesthesia, obesity, and drugs (2). Brain injuries and head traumas are also among the most important causes of nausea and vomiting in the people admitted to the emergency department, which occur as a result of occupational and traffic accidents. In Iran, traffic accidents are a major cause of traumatic injuries (3, 4). According to studies, the frequency of nausea and vomiting in people with trauma varied from 10% to 60% (5, 6). The prevalence of nausea and vomiting was also higher in people with head traumas than other traumatic events and was reported to be about 25% to 30%. The nausea and vomiting after traumatic events even increased the risk of skull fractures by 4 times (7). 

Nausea and vomiting are considered as two warning signs of head trauma, which increase the risk of aspiration and intracerebral pressure in hospitalized patients, threatening their lives (6). On the other hand, due to the suffering of the patients and the possible injuries caused by nausea and vomiting, sedation and treatment in the emergency department is of potential importance (8).

Antiemetics are among the most common drugs prescribed to treat the patients with nausea and vomiting in the emergency department (9). Ondansetron is one of the most important drugs for the treatment of nausea in the people with gastrointestinal disturbances and those undergoing chemotherapy and surgery. It is not influenced by dopamine receptors and thus, has no extrapyramidal side effects (10). Ondansetron is intermittently prescribed to children and infants due to its beneficial efficacy in reducing nausea in addition to its lack of extrapyramidal side effects, so in 2008 ,it was recognized as one of the 200 highly prescribed drugs (8, 11). Metoclopramide is also one of the most common drugs for controlling and preventing nausea and vomiting, but it is associated with extrapyramidal effects due to binding to dopamine receptors. Therefore, there is always a precautionary measure in prescribing it (12-14). 

Given that nausea and vomiting are among the most common complications of mild head trauma and cause increased restlessness and harassment as well as increased brain pressure and aspiration, treating them can increase patient’s comfort and prevent further complications such as dehydration, hypokalemia, and aspiration (9, 15). Considering the limited studies on the effectiveness of ondansetron and metoclopramide in controlling nausea in the patients with mild head trauma, this study aimed to compare the effectiveness of treatment with these two most common anti-nausea drugs in patients with nausea and vomiting caused by mild head trauma in the emergency department. 

## Methods

This randomized double-blind placebo-controlled clinical trial was carried out in the emergency department of Shahid Hasheminejad Hospital in Mashhad during April 2017 to June 2018 after adopting the ethical license from the organizational ethics committee (code of ethics: IR.MUMS.fm.REC.1395.642). All the patients referring to the emergency department with a mild head trauma complaint who met the inclusion criteria such as nausea and vomiting, normal brain CT scan, triage level of 3≤, not taking antiemetic drugs within 8 hours before hospitalization, and injury time of <24 hours entered the study once their written informed consent was obtained.

The exclusion criteria were traumas to other parts of the body (especially the abdomen), chronic diseases (hypertension, diabetes, heart disease, and malignancies), persistent vomiting 1 hour after hospitalization, alcohol consumption, unstable hemodynamics, allergies, drug allergies to metoclopramide and ondansetron, and GCS 13 & 14. The patients were classified into the following three groups using convenience sampling and random allocation approaches (through the use of the random digits table): metoclopramide, ondansetron, and placebo treatment. 

They were fully examined and their basic information as well as their complaints about nausea and vomiting were recorded in a checklist designed in advance. The subjects received ondansetron (4mg/4ml, as a slow intravenous injection), metoclopramide (10 mg / 4 ml, as a slow intravenous injection), and a placebo (4 ml saline solution). All the injections were administered by emergency medicine specialists. The drugs were prepared by the researchers in similar numbered syringes according to the three groups, and the injectors and the participants were blind to the nature and effects of the drugs. The patient complaint of nausea and vomiting was recorded in the study checklist 15 and 30 minutes after the injection. The severity of nausea was assessed using the VAS (visual analogue scale) self-report scale before the injection and 15 and 30 minutes after it.

By default, VAS is a 100-mm marker, the left and right of which indicate the lack of nausea and a higher intensity of nausea, respectively. The changes greater than 20 mm in nausea have been shown to be clinically significant. The severity of nausea was divided into three groups based on the VAS scale: 1- severe nausea (VAS> 70 mm), 2- moderate nausea (50 <VAS <70 mm), and 3- mild nausea (VAS <50 mm), the validity of which had been proven in previous studies (6, 16, 17). The patients' satisfaction with the treatment as well as the side effects and the need for rescue medication were also assessed in all three groups.

This study was registered in the Iranian Registry of Clinical Trials on 2018/04/15 with the code IRCT20170609034403N3.

**Statistical Methods:** Descriptive statistical methods such as mean and standard deviation were used to describe the data. The χ^2 ^test was also used to evaluate the frequency distribution of the studied variables according to the treatment groups. In addition, ANOVA and repeated measures were used to examine the mean differences of the quantitative variables and to compare the change trends in the groups, respectively. The researchers also used the percentage change to evaluate the VAS score at each time compared to the scores before treatment in the study groups. The percentage change in each group was calculated through formula 1 in which $\overset{\text{®}}{x}$ showed the value ​​of an intended variable at the beginning of the study and $\overset{\text{®}}{Y}$represented the value ​​of the variable in the second follow-up.

Equ 1: Percent Change %=x®-Y®x®×100

The statistical analysis was performed using the SPSS software (Version 16), and the significance level in this study was considered <0.05.

## Results

A total of 124 patients with mild head trauma, nausea, and vomiting who referred to the emergency department participated in the study with their written informed consent, of whom 57.26% were males and 42.74% were females. The mean age of the subjects was 32.5±12.9 years. The sex distribution in the intervention groups also showed that 56.82% and 69.23% of the subjects in the metoclopramide and placebo groups were males, respectively, and the frequency of females in the ondansetron group was higher than the other groups (53.65%). The mean systolic and diastolic blood pressures of the patients were 121.3±16.5 mmHg and 75.7±9 mmHg, respectively. In addition, the mean axillary temperature of the patients was 36.7± 0.49 C and the mean PR (pulse rate) was 81.3. 8.2 per minute (table 1). 

The mean VAS score of the patients was 68.3±29.5 mm before treatment, with no significant difference in the study groups (P=0.33) (table 1). The scores change to 38.9±23 mm and 31.7±24.2 mm fifteen and thirty minutes after the injections, respectively, showing that the mean VAS scores in the study groups were not significantly different (p> 0.05) (table 2).

**Figure 1:**
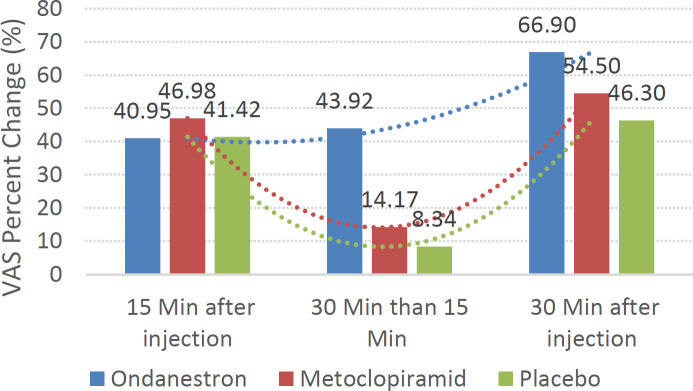
Percentage change (PC) of VAS score at each time compared to the pre-treatment time in the study groups

The percentage change (PC) of the VAS scale showed that in the first 15 minutes after the injections, the subjects treated with metoclopramide had the highest percentage change (46.97%) compared to the pre-intervention time, but the patients treated with ondansetron had the greatest changes in the mean VAS (43.94%) 15 to 30 minutes after the injections. The results of the percentage change of the VAS score within 30 minutes compared to the pre-injection time also showed that ondansetron caused the most changes in the mean VAS score (about 66.9%). Finally, the placebo group had the lowest VAS percent change at different times (figure 1). 

**Figure 2:**
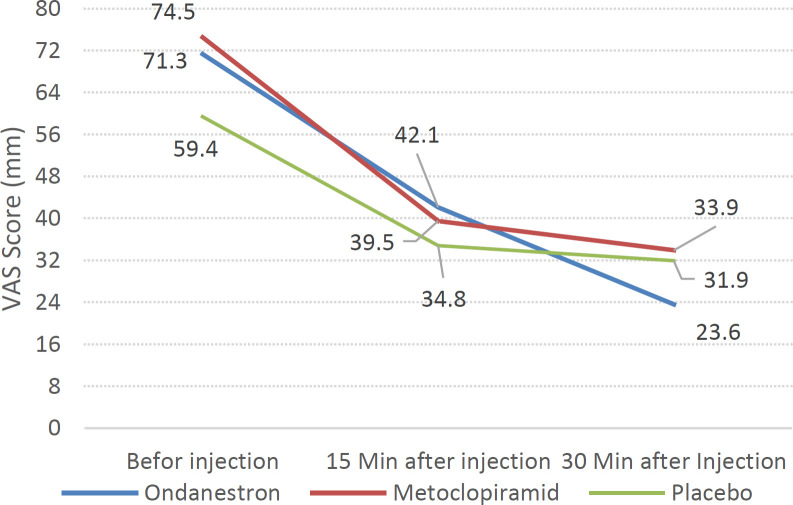
Trend of changes in VAS score in the ondansetron, metoclopramide, and placebo groups

The results of examining VAS score changes during the study showed that the mean VAS scores of the study groups treated with metoclopramide, ondansetron, and placebo decreased significantly 15 minutes and 30 minutes after the treatment (p<0.01) (table 2) (figure 2). Regarding the side effects, akathisia was not observed in any patients at any time (15 and 30 minutes after the treatment). Similarly, no patient had extrapyramidal symptoms and a headache at any time, either.

VAS: Visual analogue scale (mm).

**Table 1:** Basic and background information of the people treated with ondansetron, metoclopramide, and placebo

P- value	Total (n=124)	Intervention Groups			Group
		Placebo (n=39)	Ondansetron (n=41 )	Metoclopramide (n=44)	
**0.578**	32.5±12.9	34.2±8.2	31.7±6.9	31.5±7.3	Age (year)
**0.113**	53(42.74%)	12 (30.77%)	22 (53.65%)	19 (43.18%)	Gender (Female)
**0.150**	121.3±16.5	117.4±15.5	120.6±13.1	125.4±19.5	SBP (mmHg)
**0.110**	75.7±9	73.8±9.3	75.5±9.5	77.7±8	DBP (mmHg)
**0.510**	81.3±8.2	80.6±7.1	85±5.6	78.3±9.9	PR (Per Min)
**0.470**	36.7±0.49	36.7±0.5	36.8±0.4	36.6±0.4	Axillary Temperature (C^0^)
**0.330**	68.3±29.5	59.4±23.6	71.3±19.5	74.5±35.3	VAS Score (mm)

SBP; Systolic blood pressure, DBP; Diastolic blood pressure, PR; Pulse rate, VAS; Visual analogue scale

**Table 2:** Mean VAS score of the individuals treated with ondansetron, metoclopramide, and placebo

P- value*	Intervention Groups			Group
	Placebo (n=39)	Ondansetron (n=41 )	Metoclopramide (n=44)	
**0.330**	59.4±23.6	71.3±19.5	74.5±35.3	VAS Score Before Injection (mm)
**0.460**	34.8±26.4	42.1±19.9	39.5±22.5	VAS Score 15 Min after Injection (mm)
**0.570**	31.9±27.1	23.6±25.79	25.9±21.8	VAS Score 30 Min after Injection (mm)
	0.01†	0.01†	0.01†	P-value Trend**

* P values from Analysis of Variance between Groups (ANOVA) ** P value from Repeated Measure Analysis.

† Significantly less than 0.01

## Discussion

The aim of the present study was to evaluate and compare the effectiveness of ondansetron and metoclopramide in controlling nausea in the patients with mild head trauma in the emergency department, and the results clearly showed that the two drugs were sufficiently effective in controlling nausea. The VAS score of the patients in the metoclopramide group increased before the injection within 15 and 30 minutes after the injection with 10 mg of intravenous metoclopramide in 4 ml of saline solution. In the ondansetron group, the VAS score increased before the injection within 15 and 30 minutes after the injection with 4 mg of intravenous ondansetron in 2 ml of distilled water. The VAS score of the placebo group, which had received 4 ml of intravenous saline solution, similarly reduced significantly within 30 minutes, but the percentage change was lower than that of the other study groups. In the first 30 minutes, after the injection, the VAS score of the ondansetron-treated group had the highest percentage change compared to the pre-injection time. In other words, the patients' VAS score reduced by 66%, indicating the longer half-life of this drug compared to the others. Although the patients' VAS score in the metoclopramide group decreased by about 54.49% thirty minutes after the injection, the greatest and fastest effect on the VAS score was observed fifteen minutes after the injection due to the three-minute intravenous absorption and 15-minute peak effect of the drug (6, 18, 19). Similar studies had compared the antiemetic effects of ondansetron and metoclopramide (20, 21). Zamani et al. (6) showed in their study that administration of either ondansetron or metoclopramide could significantly reduce nausea and vomiting in trauma patients. The researchers did not find any differences in the effectiveness of ondansetron and metoclopramide, and the two drugs had similar anti-nausea effects, which is consistent with the results of the present study. 

On the other hand, Zamani et al. (6) mentioned the side effects such as drowsiness and anxiety in the patients treated with metoclopramide, which were not observed in any of the subjects in the present study. In their study, Warburton et al. (19) concluded that metoclopramide, ondansetron, and placebo could similarly reduce the rate of nausea and vomiting in patients and there was no significant difference in the effectiveness of these three drugs. This is consistent with the results of the present study. Barrett et al. (15) showed that ondansetron, metoclopramide, and promethazine were none superior to placebo in terms of reducing nausea and vomiting. However, the rates of nausea and vomiting in all groups reduced significantly after 30 minutes, both statistically and clinically. It was found out in the present study that although there was no difference in effectiveness between ondansetron and metoclopramide compared to the placebo, the percentage changes of the VAS score reduction differed from the pre-injection in the three groups. 

It means that metoclopramide was relatively superior within the first 15 minutes, but the percentage change of the VAS score was higher in the ondansetron group thirty minutes after the injection, showing the effectiveness and shelf life of the drug in reducing nausea. The results of this study showed well that although there was a significant difference between ondansetron and metoclopramide in reducing nausea and vomiting in patients with mild head traumas, the percentage of VAS score changes at different times indicated the relative superiority of ondansetron in reducing the patients' nausea within the first 30 minutes of the study. However, more studies are needed to evaluate the efficacy and effectiveness of anti-nausea drugs in the emergency department.
